# Uncertainties, Fear and Stigma: Perceptions of Zika Virus among Pregnant Women in Spain

**DOI:** 10.3390/ijerph17186643

**Published:** 2020-09-11

**Authors:** Elena Marbán-Castro, Ana Villén-Gonzalvo, Cristina Enguita-Fernàndez, Anna Marín-Cos, Clara Menéndez, Maria Maixenchs, Azucena Bardají

**Affiliations:** 1 ISGlobal, Hospital Clínic—Universitat de Barcelona, 08036 Barcelona, Spain; ana.villen.gonzalvo@gmail.com (A.V.-G.); cristina.enguita@isglobal.org (C.E.-F.); annamarin1986@gmail.com (A.M.-C.); clara.menendez@isglobal.org (C.M.); maria.maixenchs@isglobal.org (M.M.); azucena.bardaji@isglobal.org (A.B.); 2 Consorcio de Investigación Biomédica en Red de Epidemiología y Salud Pública (CIBERESP), 28029 Madrid, Spain; 3 Centro de Investigação em Saúde de Manhiça (CISM), Maputo 1929, Mozambique

**Keywords:** views, perceptions, attitudes, zika, pregnancy, migrants, travelers, qualitative, grounded theory, Spain

## Abstract

Similar to other epidemics, knowledge about Zika virus (ZIKV) relies upon information often coming from outside the health system. This study aimed to explore views, perceptions and attitudes towards ZIKV among migrant women from Central and South America, diagnosed with ZIKV infection during pregnancy, and to comprehend healthcare professionals’ perceptions of ZIKV. An exploratory qualitative study, based on phenomenology and grounded theory, was conducted in Barcelona, Spain. Data were collected through in-depth and paired interviews with women diagnosed with ZIKV infection during pregnancy, and semi-structured interviews with healthcare professionals. Women showed good level of awareness of ZIKV, despite some knowledge gaps. The most consulted source of information about ZIKV was the Internet. Women expressed they suffered from anxiety and depression due to potential effects of ZIKV on their babies. They conveyed their sources of support came primarily from their partners and relatives, as well as healthcare professionals. This study stresses the dramatic health, social and emotional burden that the epidemic imposed on migrant women infected with ZIKV during pregnancy. These results may help guide psychosocial support and health measures for pregnant women and their children as part of the public health emergency response in emergent epidemics.

## 1. Introduction

Zika virus (ZIKV) was first identified in the Zika forest in Uganda in 1947 [1]. In 2015, an outbreak of ZIKV rapidly spread through The Americas and multiple other countries worldwide [2]. ZIKV is transmitted by mosquito bites, sexual intercourse, blood transfusion, organ transplantation, blood productions and during pregnancy from women to fetuses [1,3]. In most cases, ZIKV infections cause a mild self-limited disease, with skin rash and fever as its main clinical features [2], similar to other arboviral infections caused by dengue and chikungunya, endemic in Central and South America and transmitted by the same vector (*Aedes* mosquito species) [4]. ZIKV infection can cause neurological disorders such as meningoencephalitis or Guillain–Barré syndrome; and if the infection occurs during pregnancy it can be associated with spontaneous abortions, stillbirths and children with neurological impairments, known as Congenital Zika Syndrome [1,5].

In endemic areas, given the lack of availability of vaccines or drug-based treatments for ZIKV [6], recommendations from public health authorities primarily rested upon interventions to reinforce individuals’ prevention strategies to avoid human-vector contact [7]. These included indoor residual insecticide spraying, bed nets and mosquito repellent use, as well as behavioral change strategies (e.g., minimal skin exposure or condom use) [7]. In addition, some countries strongly promoted reproductive health services and increased access to ZIKV testing [7]. Following WHO declaration of the ZIKV epidemic as a Public Health Emergency of International Concern in 2016, country-specific recommendations in non-endemic areas were made available to pregnant women and women of reproductive age including advice to avoid traveling to affected countries, the use of condoms with partners returning from endemic areas or to postpone pregnancies up to two years [8]. To date, ZIKV is no longer considered as an emergency but a common threat, even though more than 70 countries still report active transmission of the virus [9].

News and mass media played an important role in the dissemination of information to the general public during the ZIKV epidemic [10]. The information that was shared outside the health system was often of poor quality and rarely based on verified sources [10]. News and information can influence knowledge, risk perceptions and ways to prevent the infection. A study in Florida found that women with higher knowledge about the ZIKV were nearly six times more likely to uptake preventative measures to avoid ZIKV infection [11]. That study highlighted that different risk perceptions among patients and healthcare professionals might influence attitudes towards proposed measures for controlling the disease [11]. Messages advocating to avoid pregnancies overlooked economic, structural and social barriers women face in countries in Central and South America [12,13], where structural gender inequities are well documented decision factors [14]. Moreover, women living in ZIKV endemic areas often have limited access to reproductive health services [15].

There are several studies reporting perceptions and knowledge gaps in pregnant women exposed to ZIKV in endemic areas [12,16,17,18,19]; access to the healthcare system, specifically to sexual and reproductive health services for women living in endemic settings [20,21,22]; perceptions of ZIKV among women, not necessarily during gestation [9,23]; the general public living in endemic settings [11]; knowledge and attitudes among travelers to the U.S.A. [24] and to Brazil [25]; and perceptions of ZIKV among healthcare professionals [26]. The previously mentioned studies are very diverse in design, methodology and population under study, as are in their results and conclusions. To our knowledge, there are no studies on perceptions of ZIKV among migrant women in a non-endemic country, such as Spain. Also, few studies have addressed the psychosocial implications of the ZIKV epidemic on women who might be considered to be affected to a lesser extent by the epidemic [9], such as women diagnosed with ZIKV during pregnancy and who delivered healthy children, even though they still suffered from ZIKV related fears and uncertainties.

Qualitative research studies on populations affected by the ZIKV epidemic help better understand community needs and guide public health outreach practices. Sociocultural epidemiology, as the context in between epidemiology and medical anthropology, is a key area to stress the importance of personal, social, economic and political processes that may influence populations’ health and to further understand established relationships among healthcare workers and patients [27]. This study aimed to explore views, perceptions and attitudes towards ZIKV, and its preventive and control measures, among migrant women from Central and South America living in Spain who traveled to their countries of origin during the epidemic and were diagnosed with ZIKV infection during pregnancy; and to comprehend healthcare professionals’ views, perceptions and attitudes while communicating ZIKV information.

## 2. Materials and Methods

### 2.1. Study Site and Population

The study was conducted in Barcelona, Spain, between September 2018 and February 2020. Participants were identified from a surveillance study of ZIKV during pregnancy at the Institut Clínic de Ginecologia, Obstetrícia I Neonatologia (BCNatal), a consortium of the Hospital Clínic Barcelona (HCB) and Sant Joan de Déu (SJD) Barcelona Children′s Hospital. Study participants were women diagnosed with ZIKV infection (confirmed or probable cases) during pregnancy and a subset of healthcare professionals who assisted these women during pregnancy and childcare. Pregnant women with a confirmed or probable ZIKV diagnosis were followed up from recruitment until delivery. Their children were followed up to two years of age.

Inclusion criteria for study participants were defined as having traveled to an endemic area for ZIKV during pregnancy; attending to antenatal care at BCNatal; having a confirmed result for ZIKV during pregnancy (positive ZIKV-PCR) or a probable ZIKV infection during pregnancy (positive serological analysis with a negative PCR result); and who were willing to be interviewed. Inclusion criteria for healthcare professionals were defined as having provided clinical or preventive care to pregnant women who had traveled to ZIKV endemic areas and/or to their children. Invited healthcare providers included obstetricians, pediatricians and tropical diseases’ specialists.

A minimum sample size of 15 participants was considered, according to previous study experiences in reaching the saturation point and theoretical saturation [28,29,30]. Saturation point was defined as whereby all themes had been thoroughly explored in detail and no new themes kept emerging in subsequent data collection [28,29,30]. Saturation could be obtained before or after the minimum sample size was reached.

### 2.2. Study Design

This was an exploratory qualitative study based on phenomenology and grounded theory. Grounded theory is an inductive approach where theoretical generalizations emerge from the data rather than being assumed beforehand [28]. Phenomenology is an approach to understand the first-hand experiences of those involved in a phenomenon of interest, and it is particularly useful to examine complex and sensitive topics [29].

### 2.3. Data Collection

Data were collected through in-depth interviews (IDI) and paired interviews (PI) [29,30,31] for women exposed to ZIKV during pregnancy (confirmed and/or suspected cases) and semi-structured interviews (SSI) for healthcare professionals. IDIs were used to collect detailed information about women’s experiences and perceptions and to explore new topics in depth [29]. PIs, also known as mini focus groups [31], were used for the purpose of collecting information about how small groups perceive an event, by sharing information among women having experienced similar situations and recalling memories from others [30,31,32]. SSIs were performed with healthcare professionals to maximize data collection, as main topics already arose from previous interviews with women, for optimizing responses to specific topics, and for healthcare staff time concerns. Data collection took place from December 2018 to February 2020. Interviews were carried out at the place of preference of the participants, including health facilities, participants′ place of residence, or public places. PIs took place in a room at HCB. All interviews were digitally recorded and notes were taken. Researchers came together regularly to discuss key findings, difficulties and any appropriate change to the data collection guides, according to the data that emerged. All interviews were transcribed anonymized and data were double transcribed and coded for quality control purposes.

### 2.4. Data Analysis

Data were coded using Dedoose^®^ software (SocioCultural Research Consultants, LLC, Manhattan Beach, CA, USA) and consensus on codes and emerging themes was reached in meetings within the research team. Grounded theory was used as a methodological and analytical approach. Research began with no pre-existing hypothesis, allowing a theory to inductively emerge from the data, following a systematic and circular data collection and analysis. Theory generation was based on comparative analyses among data collected from different participants, and pre-existing conceptualizations were not used [29].

### 2.5. Ethical Considerations

Ethical approval for the study was granted by the Ethics Review Committee of the HCB, Barcelona, Spain (CEIC) (Reg. No. HCB/2016/0250). The study was conducted in accordance with the Good Clinical Practice Guidelines and under the provisions of the Declaration of Helsinki and local rules and regulations. Participants gave written and oral consent for interviews and audio recording. All names in the transcripts were deleted to guarantee subject anonymity. Data were analyzed anonymously.

## 3. Results

### 3.1. Participants′ Profile

Women with confirmed or probable ZIKV infection during pregnancy, according to laboratory diagnosis, participated in IDIs (*n* = 12) and PIs (*n* = 5). Gestational age at ZIKV diagnosis was different for each woman depending on their arrival from country at risk and time of ZIKV analysis. Three women were pregnant at the time of the interview and 14 delivered healthy babies. Five of these children presented a slight language delay at two years of age, when the interview occurred. The average age of women was 31 years old (range 22–42 years). The average time living in Spain was 8.4 years, with a minimum period of 1 year and a maximum of 18 years. More than half the participants (10/17) reported being unemployed and being main caregivers for their children. Two thirds of study women declared to be Christians. Healthcare professionals enrolled included three obstetricians, two pediatricians and one tropical medicine and international health specialist. All healthcare providers were women. The duration of IDIs was around 45–60 min, PIs 1 h 15 min and SSI lasted 30 min. Table 1 describes the sociodemographic characteristics of study participants.

Topics raised in all interviews were perceptions, reactions and feelings about ZIKV, discussions about the termination of pregnancy and stigmatization of the disease.

### 3.2. Perceptions of ZIKV

Women were asked to say the first thing that came to their minds when hearing the word “Zika”. Most of them first mentioned “*mosquitoes*”, followed by “*fear*”, “*symptoms*” (or absence of them), “*disease severity*” and “*endemic countries*”. According to the perception of ZIKV causing a mild disease, some of the participants hesitated about having been infected with ZIKV; they thought they experienced dengue or chikungunya during pregnancy and/or years before. Moreover, some participants were confused about “Zika” being a virus, a mosquito or the same disease as dengue or chikungunya. Two participants compared their symptoms with those experienced after an international journey (jet-lag); and one woman alluded that ZIKV was caused by “*the spread of an insecticide by the Monsanto company”*. Almost all participants correctly named the most common symptoms of ZIKV infection. Few respondents said that ZIKV does not give any physical presentation. Symptoms were frequently compared to those of dengue and chikungunya infection without prompting from the interviewer. In general, it was said that ZIKV symptoms were supposed to be milder. The following quotation of one participant typifies the description of the differences:

“*There [home country] Zika didn’t get so much attention because it wasn’t as painful as Chikungunya [...] It was like, well, Chikungunya!! [exclaiming] ‘Well, [people say that] Zika causes two little things and that’s all’. For them, it was two little things… but the bad thing was what was driven for pregnant women*”.

(The Dominican Republic, 42 years old, 18 years in Spain)

The majority of respondents mentioned that mosquitoes were the principal route of infection. Some of them acknowledged that not all mosquitoes transmit ZIKV and explained differences in mosquito habits as “*the mosquito of Zika only bites in the afternoon, only in the afternoon, and bites from the waist down. It does not fly up. It flies down*” (Brazil, 36 years old, 11 years in Spain). One woman explained that health interventions to control ZIKV mainly focused on individual-level behavioral changes and expressed her concern that ZIKV could not be prevented through individual actions alone, as vector control actions were beyond her own personal capacity.

“*You can’t leave standing water… the water for the dogs has to be changed 3 times per day! Because if you don’t do it, the mosquito throws the larvae there and so the mosquito reproduces there. So there [home country] people try hard not to leave things around. Even in a bottle cap [mosquitoes] can put eggs... So, you have to be very careful [...] But, I take care, my neighbor does not, there is where it grows! That’s it!*”

(Brazil, 36 years old, 11 years in Spain)

Fewer than half of the interviewees were aware that the infection can be transmitted through sexual contact, and respondents who were aware of the sexual transmission did not use condoms because they did not understand the reason, as “*the baby is already formed*”. Healthcare professionals expressed that they advised patients to use condoms during pregnancy, but they recognized this was a very unpopular measure and thought that patients did not consider this recommendation.

“*The measure given is ‘Condom use during the whole pregnancy’. There is not another possible measure [...] And I think that women’s compliance is… 0%? I mean, I think that no woman does it.*”

(Healthcare professional, number 2)

Table 2 illustrates preventive measures raised by women during interviews categorized according to routes of ZIKV transmission.

While most women knew that there is not a specific treatment for ZIKV infection, some participants declared to have received “*injections and vaccines*” for ZIKV. One participant clearly explained that “*vaccines and treatments arrived in her country*”. The majority of respondents reported symptoms relieved after self-medication of non-prescribed painkillers, mainly named “*acetaminophen*”, “*paracetamol*” or “*ibuprofen*”. Traditional remedies were used, such as coconut juice to keep hydrated and reduce fever, and a herbal infusion used in home country with tree leaves.

Some women only realized their perception of risk when someone close to them was infected; then, they started to use preventive measures. Women expressed that, despite knowing about the risk of ZIKV, only when they were pregnant they realized it was a critical issue. Healthcare professionals declared that the perceptions of risk and of disease severity were directly related to mass media messages. They declared that, in 2016 messages were very alarming, and now, in 2020, ZIKV is completely forgotten, thus minimizing the perception of severity, which could lead to under consultations, underdiagnosis of cases and severe consequences not detected in time.

“*I think that when the boom started, it was a health alert… and it was a lot of fear from women who could have had contact [with ZIKV], who travelled to endemic areas and so, but also from healthcare professionals there was much respect and we received referrals from women, many times women were referred even without a serology performed, like ‘This women has been to… that country, Oh my God, Zika!’ and she was referred to us. Today we are just at the opposite pole, fear of Zika has vanished.*”

(Healthcare professional, number 2)

Interestingly, healthcare professionals who attended pregnant women (gynecologists and specialists from the tropical medicine department) explained that they perceived fear and worry from pregnant women. Contrarily, pediatricians reported that mothers did not show concerns or anxiety when they were in consultation with their babies, even when mothers were explained that there could be long-term consequences of ZIKV.

“*In our mothers [women in the study] it was less [perception of risk] … because… they are mothers who have been feeling good, they have been followed up… that in very few occasions we found something [a problem in the woman/baby] ... So, you have an impression of ‘Well…’ Even… for example, a woman had a positive PCR and said ‘No, no, I don’t want anything to be performed with him [the baby], not to my baby, nor with me…’ and she did not want to continue postnatal follow up. So, if you are worried, you do the follow up. So, I think not… no… no… like I said, here in this context, there has not been… this… such worrisome in women*”

(Healthcare professional, number 4)

Regarding the perception of severity, some women declared that the only concern for ZIKV was when infection occurs during pregnancy “*The true problem with Zika is pregnancy, when one is pregnant. It’s almost not going to affect me [but the fetus]*”. Others mentioned that severe complications occur in non-pregnant adults, such as “*Guillain–Barré syndrome*” and “*death*”. Healthcare professionals agreed that the severity of ZIKV infection affects mainly during pregnancy. They noted that long-term repercussions in children exposed to ZIKV during pregnancy are unknown, as evidence is still growing.

Common patterns of perceptions that led to reactions and feelings experienced by women during pregnancy are depicted in Table 3.

Almost all women declared that their main support during pregnancy was their partner. Even though not all participants explained their diagnosis of ZIKV to their relatives “*not to worry them*”, many women declared their relatives were a fundamental support at that time. The health system was mentioned as a support mechanism due to its functioning behavior and messages of tranquility given by healthcare staff. In some cases, participants expressed spirituality (e.g., praying, assuming the ZIKV infection was God′s will) as a coping mechanism to confront the diagnosis and carry on.

### 3.3. Views and Perceptions about the Termination of Pregnancy

Women expressed they had thought about the termination of pregnancy, they had discussed it with their partners and/or they had been informed about it at some point during gestation. The main reasons leading to these discussions were feelings of worry about how their fetus was developing, consequences on the newborn after birth and children’s and families’ quality of life afterwards.

“*That is what I was struggling the most with [during my pregnancy], the disease, that the child would come sick. Because I said: ‘bringing a… a child like that into the word, I think that... from my point of view bringing him/her... to suffer’. Because once... you know, the baby could be born, indeed, and their parents are going to take care of him/her. But a moment arrives... mainly when I will not be here anymore [when I will die], basically, that I am her mother, who is going to take care of him? So then, I... always said ‘Bringing a child... it’s bad to say that I’m going to term..., abort... to terminate a pregnancy but... bringing him to suffer, I prefer to stop it on time’*”

(The Dominican Republic, 42 years old, 18 years in Spain)

A woman explained that, despite having some doubts about a possible termination of pregnancy, she did not ask questions to her healthcare professional because she felt embarrassed. Few women expressed that, at a certain point, they thought to be mentally prepared in order to take that decision if anomalies were detected.

“*I told my doctor and everybody ‘Look, I know that I am pregnant and that I have the virus. I am going to continue… till the end if I have to get there’. But I was ready, if the baby came with any disability, I was not going to continue with the pregnancy.*”

(The Dominican Republic, 42 years old, 18 years in Spain)

Women expressed that abortion was not a legal option in their countries. Some women felt shocked by the insensitivity to how termination of pregnancy was offered in specific primary healthcare centers, even before having a confirmatory diagnosis for ZIKV or a prenatal ultrasound with results showing fetal anomalies.

“*They explained it to me ... in the health center there, from [name of the healthcare unit] … also the midwife has explained it to me, she told me ‘This is positive so… what it implies… so… we’ll have to… I am going to refer you to Hospital Clínic, and so, but, start thinking that this could be an abortion, I mean, that you may be thinking about it, because maybe we will have to remove it, because… you are going to fasten your life, and so…’*”

(Guatemala, 33 years old, 4 years in Spain)

“*I went to the primary healthcare center and took some tests there, and as I was pregnant I told the doctor ‘Well, I think I had Zika virus’. There was nothing proved and the doctor looked at us and said ‘It depends on you; do you want to continue with the pregnancy [pause] or not?’ That way, without being sure of anything, she was already offering us a termination of pregnancy. And we felt [paused] extremely bad, because… who is capable of saying so? We were already worried, and a medical doctor saying so? Both of us were really bad after that. I went out of the hospital (name of the healthcare center) crying. When the doctor said that to us, (name of her husband) wanted to hit her [she laughs]. He wanted to kill her, because I was so bad… because [the doctor] was not sure. We did not have an analysis to confirm that I had had Zika virus. She did nothing. We simply went to talk to her. I was pregnant and there was suspicion that I had the virus [...] I looked at my husband, and my husband looked at me, and he said ‘Let’s go out of here’. No way! [...] I thought… ‘my goodness, it seems that I am taking hate of people!’*”

(Brazil, 36 years old, 11 years in Spain)

Interviewed healthcare professionals declared that sometimes, after maternal inquiry, termination of pregnancy was a topic of discussion within antenatal care visits. At that point, healthcare professionals tried to calm women by explaining that they would be carefully followed up and monthly monitored, and, if any anomaly was seen, they could discuss this topic again.

### 3.4. Stigmatization of Disease

Self-perception of stigma is a cross-cutting theme that appeared when talking about different topics such as time of ZIKV infection, ZIKV infection during pregnancy or children’s health, mainly related to cases of microcephaly seen in mass media. Upon arrival from Honduras, a woman explained how, after a conversation she had with a friend, she started feeling “*dirty*” as if she were going “*to infect others*”. Some discriminatory comments made women change their attitudes and reactions towards their sources of support. These feelings led to reactions such as stop trusting their friends and reducing their social support networks. Women mentioned they did not want to share the information with their friends to avoid being judged. The reason for not disclosing that they were infected with ZIKV to their family members was “*not to worry them*”. That change in behavior due to fear of stigmatization was reported by many women.

“*Nobody knew the problem that I was having over me at that moment [...] even you have your circle, it’s true that there are friends [...] That is why I have been telling you that I do not have friends… friends that… I stayed in the circle of my own family. I mean, my cousins are my friends and my sisters, and, If I need to tell something, to my cousins. If they have to tell me something, they tell me… and there we go. We stayed in that circle.*”

(The Dominican Republic, 42 years old, 18 years in Spain)

### 3.5. Contextualization

**Figure 1:**
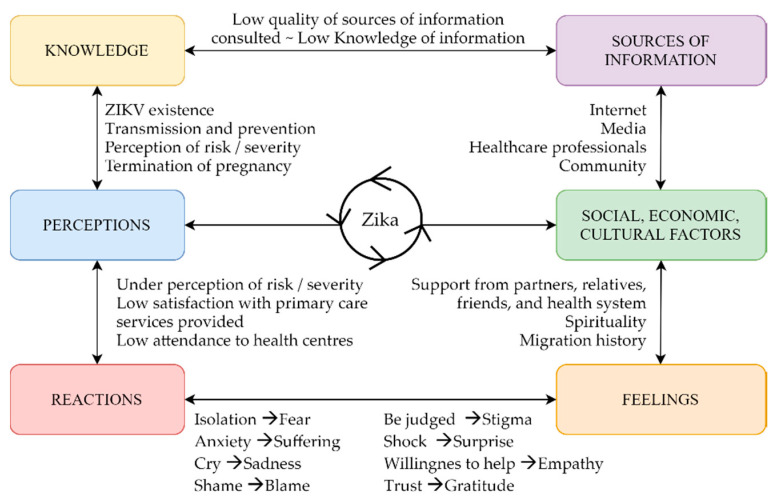
Connection of themes that arose during interviews with women diagnosed with ZIKV during pregnancy.

In general, the levels of ZIKV knowledge were related to socioeconomic determinants such as level of education or income and to the sources of information they consulted. The main source of information for participants to get informed about ZIKV was mass media, the Internet, TV, and friends and relatives living in endemic areas. Participants declared to have searched for the information on Google and YouTube, and they expressed little reliability on the information found. They confessed that information on the Internet was alarmist, incomplete and, thus, that they mostly trusted information coming from healthcare professionals. Many interviewees declared only to have actively looked for information after confirmation of diagnosis. Lack of information due to insufficient knowledge or a possible communication barrier between healthcare professionals and women led to a misunderstanding of messages such as those on preventive measures. Different levels of knowledge were translated into women’s perceptions, shaped by social, economic and cultural factors experienced during women’s lives. Perceptions were expressed in women’s reactions, such as changing medical doctors, isolation from friends and relatives and/or crying. Feelings were shaped by social, economic and cultural factors. The most relevant factors were the support received, or its lack, and migration history (experiences lived in their countries of origin and/or in Spain). Connections among these topics are illustrated in Figure 1.

### 3.6. Key Findings

- Participants showed good level of awareness about ZIKV, mainly driven by their previous experiences with similar arboviruses such as dengue and chikungunya.
- Women acknowledged the mosquito bite as the main mode of transmission of ZIKV infection, yet recognized that individual behavioral preventive measures to avoid infection went beyond their personal capacities.
- Despite overall knowledge on modes of ZIKV transmission, the majority of participants were not aware of ZIKV being a sexually transmitted infection; thus, condom use was not reported as a common practice, and those who knew about sexual transmission reported not using them, as they did not understand the underlying reasoning.
- When patients presented with symptoms, they did not attribute them to ZIKV infection, as clinical presentation was similar to those experienced with dengue or other arboviruses.
- Women were mainly concerned with ZIKV infection in pregnancy causing disability in their children; they also mentioned concerns with severe disease associated with ZIKV (e.g., Guillain–Barré syndrome) and death in adults.
- Women diagnosed with ZIKV infection during pregnancy expressed they felt fear, anxiety and even depression when thinking about potential effects for their babies’ health.
- Information disseminated on the ZIKV epidemic through mass media negatively influenced women’s feelings and emotions, impacting their emotional state.
- Internet was the most commonly used source of information about ZIKV, but at the same time a source of fear, alarmism, and misinformation that led to negative feelings.
- Misinformation about ZIKV led to stigmatization of disease, negatively affecting women, and leading to a change in their behaviors such as social isolation.

## 4. Discussion

The relevance of this study is vested in the rich in-depth information obtained on several themes raised by women who were diagnosed with ZIKV during pregnancy in Spain upon return from their countries of origin. Besides, information was triangulated by interviewing healthcare professionals who provided care to them during pregnancy and to their children after birth, and by using different data collection tools. Results show that: (1) ZIKV was mainly considered a severe disease for pregnant women and their babies and mild for other population groups; (2) women were not always aware of ZIKV being a sexually transmitted infection, and those who were aware of this route of transmission did not use preventive measures such as condoms because they did not understand the reason to use them; (3) ZIKV was shown as a stigmatized disease, and women received unpleasant messages from some friends and relatives and feared that revealing their ZIKV status could affect their social relationships; (4) coping mechanisms to deal with negative feelings, anxiety and depression derived from ZIKV diagnosis included support from partners and relatives, from quality healthcare services and spirituality; and (5) the most common source of information consulted was the Internet, although women recognized that as a source of misinformation and fear.

We found similarities between our study and a recently published article by Linde-Arias et al. [16]. Knowledge levels about ZIKV highly depend on news, access to health services and the kind of relationship with healthcare professionals; similarly, knowledge translates into perceptions that can crucially shape attitudes and practices towards preventive measures, healthcare-seeking behavior and reproductive decisions. Some women in our study reported mistaken ZIKV transmission routes (e.g., contaminated water and direct contact with a person) and preventive practices (e.g., washing hands and hygiene habits). Their reported primary source of information was the Internet, although they recognized lack of satisfaction with available data due to mistrust on the reliability of the information obtained, as similarly reported by Linde-Arias et al. [16]. Even though they were not satisfied with the information coming from the Internet, its use could be associated with the fear of stigmatization, e.g., the fear of asking specific questions to healthcare staff, friends and/or relatives. This highlights the need to strengthen trust and enhanced communications between patients and healthcare professionals to deliver accurate ZIKV information and to discuss sensitive topics, such as condom use or a possible termination of pregnancy, as well as the need to work on the de-stigmatization of the infection among the general population. Infectious-disease related stigma, such as leprosy, HIV/AIDS, tuberculosis, ZIKV, or COVID-19 could be a barrier to adopt health behaviors, leading to an increased transmission and difficulties to control outbreaks [33]. Studies have shown how Central and South American populations living in Spain experience ethnic discrimination, affecting their psycho-social health [34]. A research gap to address in future studies might be to explore knowledge and perceptions about ZIKV among local non-migrant populations as they have a lesser knowledge on arboviruses and do not suffer from ethnic discrimination.

Our findings reveal very low awareness among ZIKV infected women about the sexual route as a mode of transmission of ZIKV, and those women who had this information did not use condoms because they did not understand the reasons underlying the use of this measure, as the fetus was already developing. These results are aligned with other studies where sexual transmission was known by fewer than half of participants [12,24,35] and an analysis of the news published in the U.S.A. that revealed that almost all publications (96.8%) mentioned mosquitoes as a transmission route, but just over a half cited the sexual transmission of ZIKV (55.3%) [36]. A knowledge, attitudes and practices (KAP) analysis with American travelers concluded that knowledge of the sexual transmission of Zika significantly increases the odds of using condoms, thus improving targeted messaging through media may increase awareness and the use of preventive measures [37]. Even though participants did not completely trust the information, healthcare professionals and mass media must acknowledge ZIKV as a sexually transmitted disease [12,38]. Clear messages adapted to the general population would be important for understanding the health implications of the sexual transmission of ZIKV and a possible re-infection. In a study performed in The Dominican Republic, participants declared not using condoms because those were associated with infidelity; of note, this study was conducted in a context where women did not have the right to negotiate sexual encounters [12]. These uneven relationships could be hampering condom use in women in our study, given the similarities in population characteristics. Gender roles and norms may have influenced perceptions and implementation of preventive behaviors in our population, limiting women′s ability to initiate discussions on sexual and reproductive health with their partners [12].

Unrealistic official recommendations left women solely responsible to avoid infection; e.g., authorities recommending sexual abstinence or to postpone their pregnancies [13]. These policies are far to address the root causes of ZIKV and instead compromise women’s reproductive rights and condemn them to stigma and poorer physical, mental health and emotional outcomes [13]. It is imperative to place women and girls at the center of the discussions to understand their needs and address health issues related to ZIKV [20]. The pressure put on women impacted women’s mental health and emotional outcomes. Regarding their emotional status, we reported different feelings captured during the interviews; sadness, responsibility, shame, guilt, stigma, loneliness, sorority/sisterhood and empathy, as well as psychological suffering, anxiety and depression. Same feelings and emotions, many of them reflecting poor mental health, were reported in other studies [16,39,40]. In our study, women declared that their partners and relatives were their main source of support during pregnancy to cope with uncertainties about ZIKV, the possible transmission of infection to the fetus and repercussions on children’s health. Indeed, the legislative context influences reproductive health rights and women’s decisions [41]. Women expressed concerns about a possible termination of pregnancy and compared the context in Spain with the usual practice in Central and South America, where it is illegal in most circumstances [12,14]. The ZIKV epidemic demanded the regulatory and health authorities and the scientific community to rethink about restrictive abortion regulations in affected areas, affecting the poorest women [14] and to discuss women′s rights among the research community and policy makers [14]. Healthcare intermediaries, together with women’s knowledge and stigmatization of abortion, play a key role in women’s abortion trajectories and its access, especially in low and middle-income countries [42]. The role of gender in the epidemic and the feminization of Zika has been explained by different factors: (1) ZIKV infection disproportionately affecting women; (2) women being the primary caregivers of affected children; and (3) women being primary persons tasked with household chores to prevent infection [12]. Other factors could be added: (4) gendered economic inequities and/or gendered gap in poverty; (5) precariousness of women’s work (including unpaid work, informal work and underpaid job); and (6) pressure to adopt individual behaviors to cope with the epidemic and its impact on mental health. Addressing these topics would not only help in advancing towards the achievement of Sustainable Development Goals (SDGs) 1, 3, 4 and 5, but to manage disease outbreaks more efficiently and allow populations to live in a more equitable environment. Giving this disproportionate burden, experts have called for a gendered approach to address gender norms and health inequalities [12]. Only a feminist research agenda could challenge existing structural and social power inequalities, with an intersectional perspective, tackling race and socioeconomic inequalities, in order to advance gender equity and SDGs [43].

Healthcare professionals interviewed agreed that ZIKV messages caused alarm in 2016, having had a direct impact on the emotional state of pregnant women; and that the reduction of news related to ZIKV in 2019 and 2020 translates into an under perception of risk and low awareness about ZIKV infection and of the potential negative consequences on fetuses. Healthcare providers stressed that women had a certain knowledge about ZIKV, but not enough, and reported that patients were aware of the medical recommendations (e.g., condom use), although they were not very popular among them and thus, not used. Lastly, they concluded that sensitive topics such as a positive result in the ZIKV screening in pregnancy or any fetal anomaly should always be communicated face to face with proper counselling, empathy and sensitively.

The main limitations of the study include that results obtained may be subjected to recall bias and that the sample size may not be seen that big, although the latter does not represent a concern as saturation point was reached. The main strengths of the study lie in the feedback and insights provided directly by affected populations: women who faced the challenges of ZIKV during the 2015–2017 epidemic. Qualitative results need to be interpreted with caution, as generalizations cannot be performed. Finally, the fact that interviewees and interviewers speak the same language, as well as the interviewers being female, might have increased cooperation from participants who seemed comfortable, willing to share their experiences and participated actively in the discussions.

Based on results obtained in this study, some recommendations could be defined. Firstly, the role of healthcare professionals is key for women to get informed about ZIKV and other arboviruses. Healthcare providers, primarily those working in primary care, should reinforce information given about ZIKV and its consequences during pregnancy. Primary healthcare units are encouraged to provide psychosocial support services to maintain maternal mental health and give a space for these families to meet and exchange knowledge. Secondly, the way information is delivered must be strengthened to allow patients to freely ask questions and raise their concerns regarding sensitive topics such as confirmation of ZIKV infection, fetal disease or a termination of pregnancy. Sometimes, there are communication barriers between healthcare providers and women. These barriers need to be vanished to respond to women’s doubts with respect, empathy and considering all relevant medical information. Sensitive messages are recommended to be given face to face, with proper counselling and advice.

## 5. Conclusions

This study stresses the dramatic health, social and emotional burden that the ZIKV epidemic imposes on women infected during pregnancy by exploring views, perceptions and attitudes about ZIKV, including prevention and control measures, of migrant women living in Spain and healthcare professionals.

Women participating in this study perceived ZIKV as a terrible disease for pregnant women, leading them to overthinking of uncertainties about their children’ health. Feelings and reactions expressed included stigma about spreading the disease to other people and guilt for having been infected during gestation, which led them to isolation and emotional suffering. Women trusted healthcare professionals for receiving accurate information regarding ZIKV infection. Public health messages regarding ZIKV in general and infections during pregnancy in particular, specifically in an epidemic context, must be designed taking into consideration affected populations and information channels usually employed by them.

Pregnant women dealing with a diagnosis of ZIKV should receive medical and psychological support, including tools to improve their coping mechanisms to face the challenges (medical, psychological, social and economic) associated with the infection. These findings highlight the importance of social sciences research to provide relevant information and guide psychosocial support, clinical protocols and health preventive measures in order to respond to emerging epidemics and public health emergencies.

## Figures and Tables

**Table 1:** Demographic characteristics of women participating in the study.

Characteristics	Women Diagnosed with ZIKV during Pregnancy (Confirmed or Probable Infection) (N = 17)
**Age (years)**	
<25	3
25–35	12
>35	2
**Nationality**	
The Dominican Republic	6
Honduras	5
Colombia	2
Venezuela	2
Guatemala	1
Brazil	1
Education *	
Primary	1
Secondary	8
University or higher	6
**Occupation**	
Unemployed	10
Formally or informally employed	7
**Marital status**	
Married or living with a partner	15
Single	2

* Two missing values.

**Table 2:** ZIKV-prevention behaviors explored among participants.

Transmission Route	Preventive Measures
Mosquito bites	Skin repellent In-house repellent Wearing long-sleeve clothes Sleeping under mosquito nets Removing standing water Cleaning and covering water storage containers/adding poison there Fumigation Cutting grass
Sexual intercourse	Condom use
Maternal-fetal	Measures to avoid mosquito bites during pregnancy
Other transmission routes thought to transmit ZIKV by women in the study: Airborne transmission and close contact	Hand and face washing Avoid sleeping close to a person who is sweating Avoid close contact with people, avoid touching the skin of a sick patient

**Table 3:** Perceptions, reactions and feelings expressed by women with diagnosis of ZIKV during pregnancy.

Perceptions	Reactions	Feelings
Not expecting to be infected when they received ZIKV results	Shock	Surprise
Uncertainties of fetal complications	Anxiety attacks, higher stress levels	Suffering, fear
Do not want to share this information (ZIKV status) to avoid further questions and/or not worry others	Isolation, stop sharing information	Worrisome, fear
Uncertainties appear when women thought healthcare professionals had more information but did not want to share with patients in order not to worry them	Stop trusting healthcare professionals	Suffering, fear
Not deserving this situation, perceptions that they cannot handle it	Crying, manifest depression	Sadness
Evidence that several tests have been done during pregnancy, then the fetus is well followed-up	Trust in pregnancy follow up	Tranquility, relief
Comparing health systems (in their country of origin) and medical attention received in the current pregnancy (in Spain)	Trust in healthcare professionals	Gratitude, hope
Positive messages of support by friends and relatives as drivers of support	Talking to friends and relatives	Gratitude, relief
Perceptions that this interview/study could help others in the future; concerns about if ZIKV will continue in the future affecting women	Willingness to help other women	Empathy, sorority
Perceptions about the possible use of preventive measures to have avoided mosquito bites and/or have avoided pregnancy	Shame themselves, overthinking	Blame
Perceptions of being stigmatized or judged by other people	Shame themselves, stop sharing information	Blame
